# Description of a new species of the orb-weaver spider genus *Zangaraneus* Mi, Wang & Feng, 2026 (Araneae, Araneidae) from China

**DOI:** 10.3897/BDJ.14.e199306

**Published:** 2026-06-15

**Authors:** Zhao Wang, Cheng Wang, Xiaoqi Mi

**Affiliations:** 1 School of Life and Health Science, Kaili University, Kaili, China School of Life and Health Science, Kaili University Kaili China; 2 Biodiversity Conservation and Utilization in the Fanjing Mountain Region, Tongren University, Tongren, China Biodiversity Conservation and Utilization in the Fanjing Mountain Region, Tongren University Tongren China; 3 Guizhou Provincial Key Laboratory of Biodiversity Conservation and Utilization in the Fanjing Mountain Region, Tongren University, Tongren, China Guizhou Provincial Key Laboratory of Biodiversity Conservation and Utilization in the Fanjing Mountain Region, Tongren University Tongren China

**Keywords:** Arachnida, biodiversity, diagnosis, morphology, taxonomy

## Abstract

**Background:**

The orb-weaver spider genus *Zangaraneus* Mi, Wang & Feng, 2026 contains four species known only from Xizang, China. In our recent study, the fifth species has been found and is described herein.

**New information:**

A new species of the genus *Zangaraneus* from central China is described: *Zangaraneus zhishengi* **sp. nov**. (♂♀). Diagnostic photos of habitus and copulatory organs are provided.

## Introduction

The orb-weaver spider genus *Zangaraneus* Mi, Wang & Feng, 2026 was established by Mi et al. (2026) to accommodate four species from Xizang, China. The representatives of this genus are characterised by an epigyne with a pair of atria and a pedipalp bearing an almost triangular median apophysis and a slender terminal apophysis appendix.

The Shennongjia Mountains lie on the border between Chongqing and Hubei. This area is home to one of the most biodiverse regions in central China and boasts one of the best-preserved primary forest ecosystems. Although some spider families in the area have been studied over the past five years (Irfan et al. 2022, Irfan et al. 2023, Wen et al. 2024, Wang and Zhang 2025, Zhang and Zhang 2025, World Spider Catalog 2026), the araneid fauna of this region remains unclear. A new species of the genus *Zangaraneus* was identified amongst the araneid specimens collected from this region. This paper aims to describe the new species.

## Materials and methods

Methods mainly follow Mi et al. (2024). The specimens are deposited in the Museum of Tongren University, China (TRU). All measurements are given in millimetres (mm). Leg measurements are given as total length (femur, patella + tibia, metatarsus, tarsus). Abbreviations used in the text and figures are as follows:

ALE = anterior lateral eye; AME = anterior median eye; At = atrium; BE = broken embolus; C = conductor; CD = copulatory duct; CO = copulatory opening; E = embolus; FD = fertilisation duct; LS = lateral sclerite; MA = median apophysis; MOA = median ocular area; PLE = posterior lateral eye; PME = posterior median eye; PP = posterior plate; Sp = spermatheca; TA = terminal apophysis; TAA = terminal apophysis appendix.

## Taxon treatments

### Zangaraneus zhishengi

Z. Wang, C. Wang & Mi, 2026 sp. nov.

62427C47-4FC5-576E-A45D-2F3A411272DB

#### Materials

- **Type status:** Holotype. **Occurrence:** recordedBy: L.Y. Wang et al.; individualID: TRU-Araneidae-793; individualCount: 1; sex: male; occurrenceID: FAB8A8B0-F9A1-52DC-86D3-989F7AAA6969; **Location:** country: China; stateProvince: Hubei; locality: Shennongjia Forestry District, Hongping Township, Yazikou; verbatimElevation: 1780 m; verbatimLatitude: 31°30.90'N; verbatimLongitude: 110°20.04'E; **Event:** year: 2023; month: 9; day: 21
- **Type status:** Paratype. **Occurrence:** recordedBy: L.Y. Wang et al.; individualID: TRU-Araneidae-794; individualCount: 1; sex: female; occurrenceID: DBF5F4B3-301B-53B3-A070-AFB34BC0A1AB; **Location:** country: China; stateProvince: Hubei; locality: Shennongjia Forestry District, Hongping Township, Yazikou; verbatimElevation: 1780 m; verbatimLatitude: 31°30.90'N; verbatimLongitude: 110°20.04'E; **Event:** year: 2023; month: 9; day: 21
- **Type status:** Paratype. **Occurrence:** recordedBy: L.Y. Wang & H.Y. Chen; individualID: TRU-Araneidae-795; individualCount: 1; sex: male; occurrenceID: 763FC64D-2E8B-5465-BED7-530D85E41B81; **Location:** country: China; stateProvince: Chongqing; county: Wuxi; locality: Lanying Township, Xi'an Village, Yintiaoling National Nature Reserve; verbatimElevation: 1637 m; verbatimLatitude: 31°29.90'N; verbatimLongitude: 109°57.31'E; **Event:** year: 2022; month: 9; day: 24
- **Type status:** Paratype. **Occurrence:** recordedBy: L.Y. Wang & T.Y. Ren; individualID: TRU-Araneidae-796; individualCount: 1; sex: female; occurrenceID: A42620CC-937D-53C4-ACD9-04C1B55B1EBE; **Location:** country: China; stateProvince: Chongqing; county: Wuxi; locality: Yintiaoling National Nature Reserve, Zhuanping; verbatimElevation: 2178 m; verbatimLatitude: 31°29.90'N; verbatimLongitude: 109°57.31'E; **Event:** year: 2022; month: 8; day: 18
- **Type status:** Paratype. **Occurrence:** recordedBy: L.Y. Wang et al.; individualID: TRU-Araneidae-797; individualCount: 1; sex: male; occurrenceID: 0F0D593C-E5FB-5B37-A921-65FDF65EF3F8; **Location:** country: China; stateProvince: Hubei; county: Zhushan; locality: Duheyuan National Nature Reserve, Zhushanyazi; verbatimElevation: 1802 m; verbatimLatitude: 31°31.53'N; verbatimLongitude: 110°0.35'E; **Event:** year: 2023; month: 9; day: 19
- **Type status:** Paratype. **Occurrence:** recordedBy: L.Y. Wang et al.; individualID: TRU-Araneidae-798–799; individualCount: 2; sex: 1 male, 1 female; occurrenceID: C6FC448B-8B28-579D-8F28-AFDDBBEB3737; **Location:** country: China; stateProvince: Hubei; locality: Shennongjia Forestry District, Dajiuhu National Wet Park; verbatimElevation: 1778 m; verbatimLatitude: 31°30.48'N; verbatimLongitude: 110°0.29'E; **Event:** year: 2023; month: 9; day: 19

#### Description

**Figure 1.**
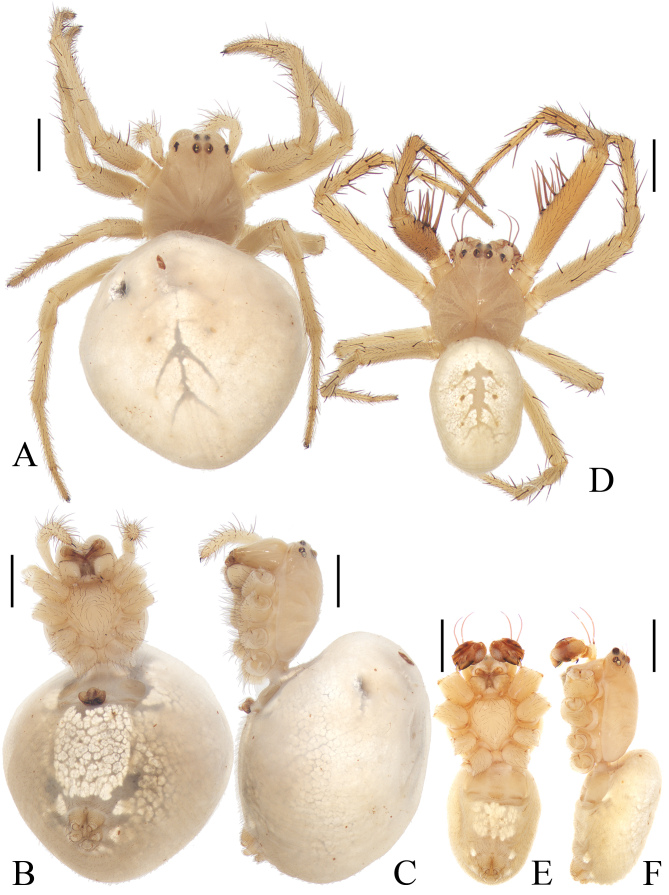
*Zangaraneus zhishengi* sp. nov., habitus. **A–C** female paratype TRU-Araneidae-794; **D–F** male holotype. Dorsal view (A, D); ventral view (B, E); lateral view (C, F). Scale bars = 1.0 mm.

**Figure 2.**
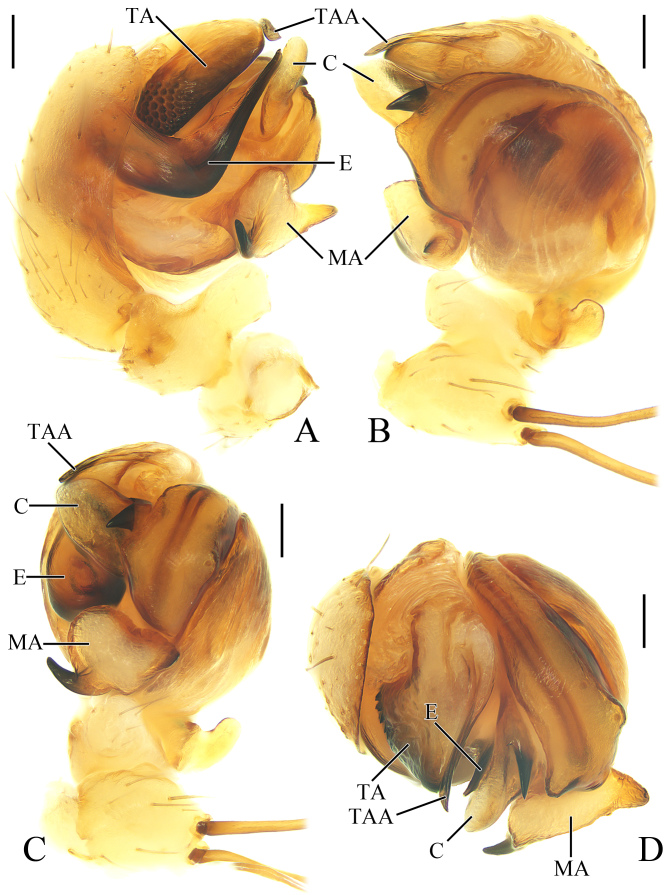
*Zangaraneus zhishengi* sp. nov., male holotype. **A** pedipalp, prolateral view; **B** *Ibid*., retrolateral view; **C** *Ibid*., ventral view; **D** *Ibid*., apical view. Abbreviations: C = conductor; E = embolus; MA = median apophysis; TA = terminal apophysis; TAA = terminal apophysis appendix. Scale bars = 0.1 mm.

Male (holotype, Fig. 1D–F and Fig. 2). Total length 4.30. Carapace 2.10 long, 1.80 wide. Abdomen 2.50 long, 1.80 wide. Clypeus 0.13 high. Eye sizes and interdistances: AME 0.10, ALE 0.08, PME 0.13, PLE 0.08, AME–AME 0.10, AME–ALE 0.20, PME–PME 0.10, PME–PLE 0.23, MOA length 0.38, anterior width 0.33, posterior width 0.35. Leg measurements: I 8.20 (2.70, 2.60, 2.00, 0.90), II 7.70 (2.60, 2.40, 1.80, 0.90), III 4.60 (1.60, 1.40, 1.00, 0.60), IV 6.30 (2.10, 2.00, 1.50, 0.70). Carapace pear-shaped, yellow. Fovea longitudinal. Chelicerae yellow, with four promarginal and three retromarginal teeth. Endites somewhat square, yellow at base, with very narrow brown anterior edge. Labium almost triangular, yellow. Sternum cordiform, yellow with brown setae. Legs yellow without annuli, femur I with 10 prolateral macrosetae at middle. Abdomen elliptical, about 1.19× longer than wide, whitish-yellow with inverted branch-like markings. Venter abdomen white, with two pairs of white spots around spinnerets. Spinnerets yellow.

Pedipalp (Fig. 2) with two patellar bristles, median apophysis wide, somewhat triangular, with slender spur at each end; embolus tapered, anti-clockwise curved about 90° near base of median apophysis; conductor membranous, rounded distally, with large, heavily sclerotised spur at base; terminal apophysis heavily sclerotised, thick, with denticles at base; terminal apophysis appendix long, flattened, weakly sclerotised.

**Figure 3.**
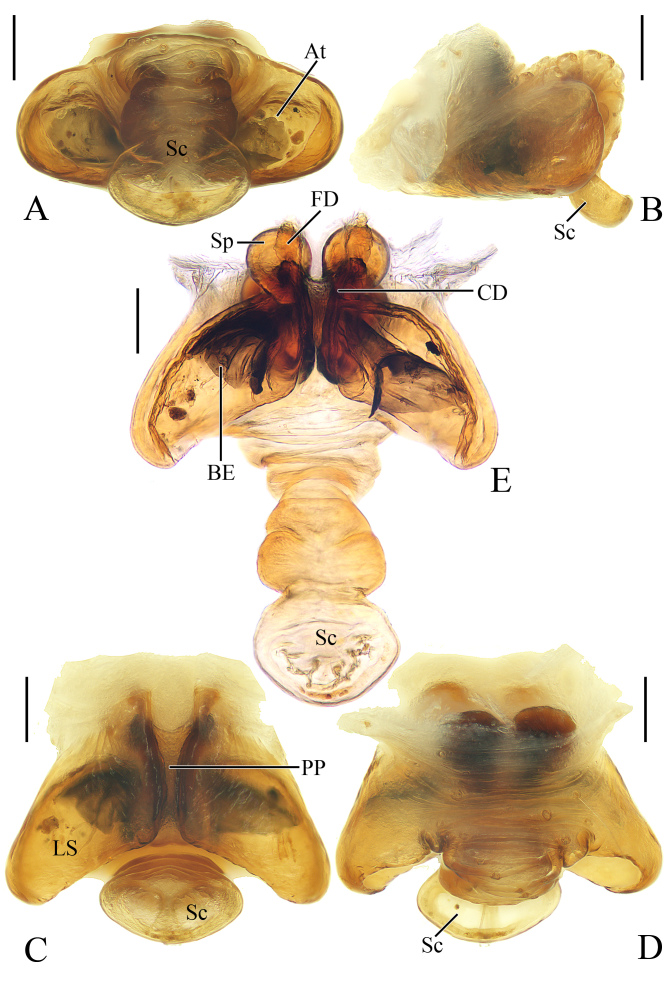
*Zangaraneus zhishengi* sp. nov., female paratype TRU-Araneidae-794. **A** epigyne, ventral view; **B** *Ibid*., lateral view; **C** *Ibid*., posterior view; **D** *Ibid*., anterior view; **E** vulva, posterior view. Abbreviations: At = atrium; BE = broken embolus; CD = copulatory duct; CO = copulatory opening; FD = fertilisation duct; LS = lateral sclerite; PP = posterior plate; Sc = scape; Sp = spermatheca. Scale bars = 0.1 mm.

Female (paratype TRU-Araneidae-794, Fig. 1A–C and Fig. 3). Total length 6.30. Carapace 2.50 long, 2.00 wide. Abdomen 4.70 long, 4.40 wide. Clypeus 0.08 high. Eye sizes and interdistances: AME 0.10, ALE 0.08, PME 0.13, PLE 0.08, AME–AME 0.15, AME–ALE 0.30, PME–PME 0.15, PME–PLE 0.30, MOA length 0.38, anterior width 0.33, posterior width 0.38. Leg measurements: I 7.00 (2.20, 2.50, 1.50, 0.80), II 6.70 (2.20, 2.30, 1.40, 0.80), III 4.70 (1.60, 1.50, 1.00, 0.60), IV 6.50 (2.10, 2.10, 1.60, 0.70). Habitus shape and colouration similar to that of male.

Epigyne (Fig. 3) with epigynal plate about 2× wider than long in ventral view; posterior plate constricted at middle part, fused with lateral sclerites; scape with many fine ridges and grooves, spoon-shaped distally, sinuous in lateral view, posterior end approximately equal to epigastric furrow; atria almost round; copulatory openings situated at inner margin of atria; copulatory ducts heavily sclerotised, thickened to spherical shaped at origin, narrower at end and almost straight in posterior view; spermatheca rounded, less than its radius separated.

#### Diagnosis

Male of the new species resembles *Z. zhui* Mi, Wang & Feng, 2026 in pedipalp structure, but can be distinguished as follows: 1) embolus lacking long embolic lamella in prolateral view (Fig. 2A) *vs*. with long embolic lamella (Mi et al. 2026: fig. 58A); 2) spur at basal conductor large, heavily sclerotised (Fig. 2B–D) vs. small, weak sclerotised (Mi et al. 2026: fig. 58B–D); and 3) abdomen lacking dark patches (Fig. 1D) vs. with five pairs of dark patches posteriorly (Mi et al. 2026: fig. 57A). Female of the new species resembles *Z. liui* Mi, Wang & Feng, 2026 in epigyne structure, but differs in: 1) outline of atrium almost round (Fig. 3A) vs. ear-shaped (Mi et al. 2026: fig. 51A); 2) anterior surface of epigynal plate procurved in ventral view (Fig. 3A) vs. recurved (Mi et al. 2026: fig. 51A); 3) copulatory ducts almost straight in posterior view (Fig. 3E) vs. spiral (Mi et al. 2026: fig. 51E).

#### Etymology

The specific name is a patronym of arachnologist Prof. Zhisheng Zhang (Southwest University, Chongqing); noun (name) in genitive case.

#### Distribution

China (Chongqing, Hubei).

## Supplementary Material

XML Treatment for Zangaraneus zhishengi

## References

[B14218252] Irfan M., Wang L. Y., Zhang Z. S. (2022). Two new species of Micronetinae Hull, 1920 spiders (Araneae: Linyphiidae) from Yintiaoling Nature Reserve, Chongqing, China. Acta Arachnologica Sinica.

[B14218261] Irfan M., Wang L. Y., Zhang Z. S. (2023). One new genus and nine new species of Linyphiidae spiders from Yintiaoling Nature Reserve, Chongqing of China. Zootaxa.

[B14216708] Mi X. Q., Wang C., Li S. Q. (2024). Description of six new genera and twenty species of the orb-weaver spider family Araneidae (Araneae, Araneoidea) from Xishuangbanna, Yunnan, China. Zoological Research: Diversity and Conservation.

[B14216717] Mi X. Q., Wang C., Feng Z. G. (2026). Description of three new genera and twenty-one new species of the orb-weaver spider family Araneidae (Araneae: Araneoidea) from Qomolangma National Nature Reserve, Xizang, China. Zoological Systematics.

[B14218270] Wang L. Y., Zhang Z. S. (2025). Seven new species of the spider genus *Cicurina* Menge, 1871 from Yintiaoling Nature Reserve, China (Araneae: Cicurinidae). Zootaxa.

[B14218288] Wen L. L., Li C. C., Zhong Y. (2024). One new species of *Pseudopoda* Jäger, 2000 from Shennongjia, Central China (Araneae, Sparassidae). Biodiversity Data Journal.

[B14218297] Catalog World Spider World Spider Catalog. Version 27. Natural History Museum Bern. http://wsc.nmbe.ch.

[B14218314] Zhang X. Y., Zhang Z. S. (2025). On two wolf spiders from Yintiaoling Nature Reserve of Chongqing, China (Araneae, Lycosidae). Zootaxa.

